# Use of surgical grafting as a part of multidisciplinary treatment for a patient treated with fixed orthodontic therapy to improve treatment outcomes

**DOI:** 10.1002/ccr3.8386

**Published:** 2023-12-29

**Authors:** Hoang Viet, Tran Hong Phuoc, Hoang Ming Tuyen, Anand Marya

**Affiliations:** ^1^ Department of Orthodontics Saigon Dental Private Hospital Ho Chi Minh City Vietnam; ^2^ Department of Implantology Saigon Dental Private Hospital Ho Chi Minh City Vietnam; ^3^ Department of Prosthodontics Saigon Dental Private Hospital Ho Chi Minh City Vietnam; ^4^ Department of Orthodontics, Faculty of Dentistry University of Puthisastra Phnom Penh Cambodia; ^5^ Dental Research Unit, Centre for Global Health Research, Saveetha Medical College and Hospital Saveetha Institute of Medical and Technical Sciences Chennai India

**Keywords:** diastema. Gingival grafting, multidisciplinary, surgery

## Abstract

**Key Clinical Abstract:**

Multidisciplinary treatment options can help provide good clinical outcomes if these are appropriately sequenced and carried out correctly. This case exemplifies interdisciplinary involvement to ensure the patient received an improved esthetic outcome.

**Abstract:**

The presence of anterior diastemas may compromise the esthetics of a patient's smile, causing mental, and social trauma in many patients. After careful evaluation Periodontal, surgical, and prosthodontic treatments are sometimes required to ensure successful treatment outcomes using a multidisciplinary staged treatment approach. These approaches must be carefully planned to ensure timely treatment progress. Surgical intervention must be planned at the right point in treatment to ensure adequate healing before placement of esthetic restorations.

## Graphical abstract

Use of Connective Tissue Grafting to correct gingival defects and improve orthodontic outcomes.

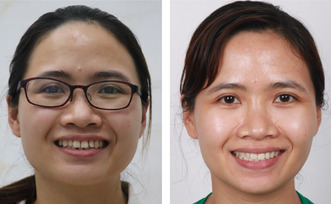

## 1 INTRODUCTION

The presence of anterior diastemas may compromise the esthetics of a patient's smile, causing mental and social trauma in many patients. In such patients' careful consideration must be given to the etiology behind the presence of the diastemas before a treatment plan can be formulated. Individual treatment planning is essential in such cases and may require more than just fixed orthodontic intervention. Periodontal, surgical, and prosthodontic treatments are sometimes required to ensure a successful outcome.^1^, ^2^

## 2 CASE REPORT

### 2.1 Diagnosis and etiology

**FIGURE 1:**
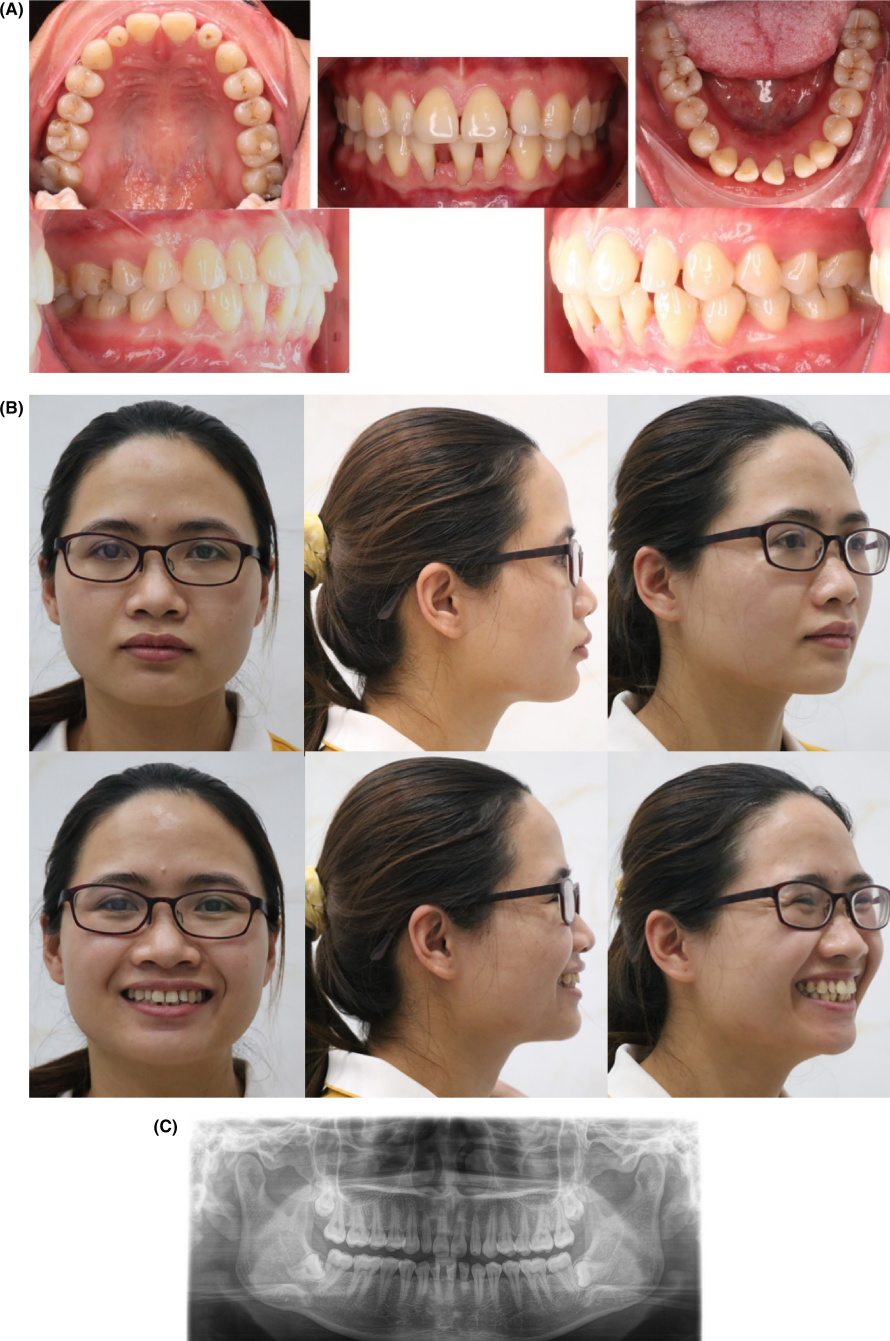
(A) Pre‐treatment Intra oral Images of the patient. (B) Pre‐treatment Extra Oral Images of the patient. (C) Pretreatment Xrays.

The patient was a 30‐year‐old woman with a chief complaint of protrusive lips, proclined. Maxillary incisors, anterior spacing, and recession on mandibular incisors. Extra‐orally, she had a convex profile with an acute nasolabial angle, a strain on the circumoral muscles while closing her mouth, and a proclination of the upper incisors was observed when the patient smiled. No symptom of temporomandibular disorders was detected (Figure 1A,B).

Intraorally, the patient was classified with a Class I molar relationship on both sides, a Class I canine relation on the right side, a Class II canine relationship on the left side, and peg‐shaped lateral incisors. The patient only had three lower incisors present, with the lower right lateral incisor missing, diastemas in the maxillary and mandibular anterior teeth because of the tongue's position, and recession on the lower incisors. The upper and lower arch forms were typically developed, and a flat Curve of Spee was observed. The patient demonstrated Bolton's tooth size discrepancies because of the three lower incisors. The upper midline coincided with the facial midline. The lateral cephalometric analysis showed a skeletal Class I jaw relationship with proclined upper and lower incisors. Both the upper and lower lips were in front of the E‐line (Figure 1C).

### 2.2 Treatment plan

The treatment plan was multidisciplinary, involving fixed orthodontics, periodontics, and prosthodontic interventions in stages. 3D simulation was planned for the patient to analyze the size of the 3 lower incisors.

### 2.3 Stage I: Fixed orthodontic therapy

**FIGURE 2:**
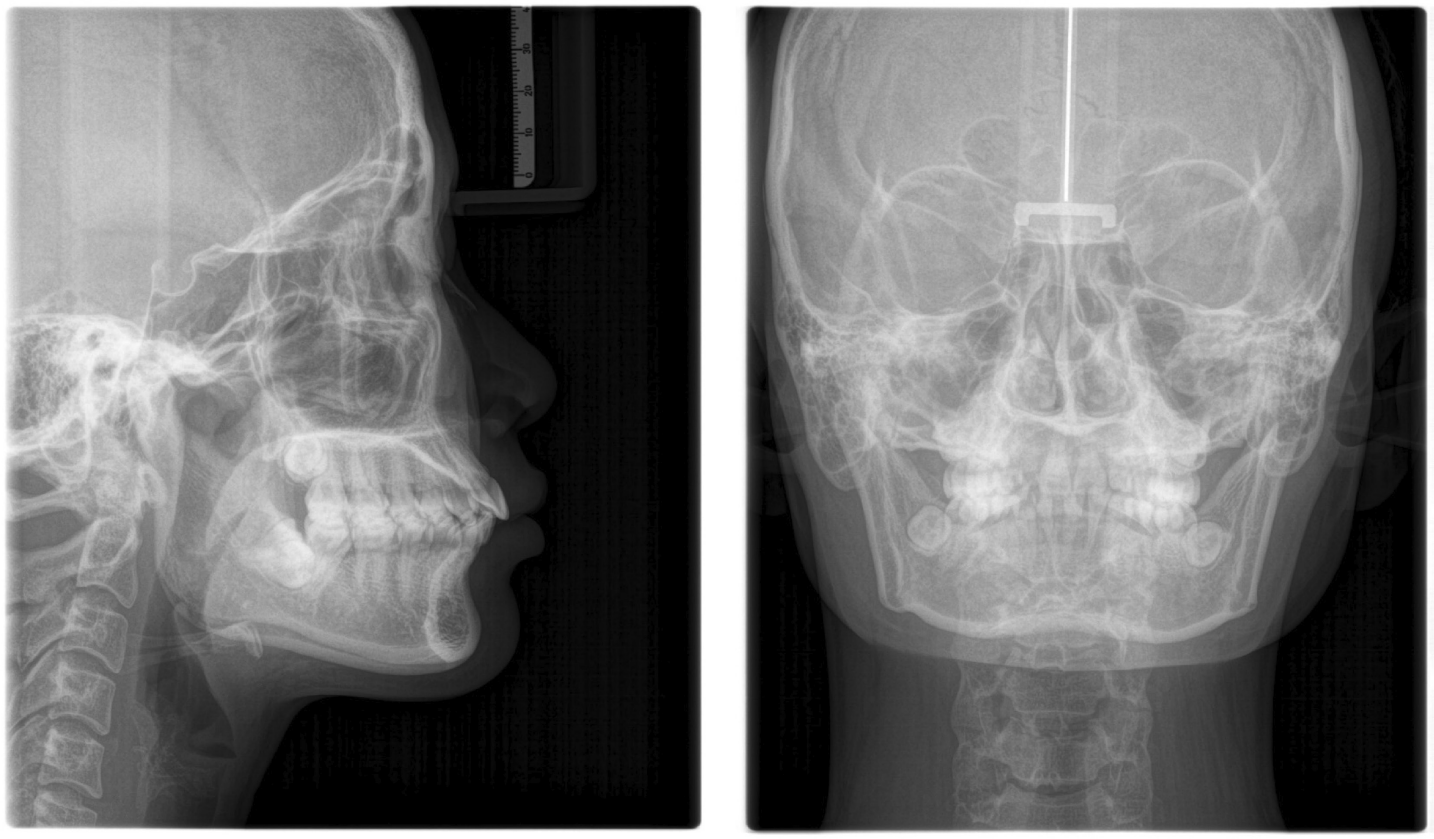
Fixed orthodontic treatment progress.

In the upper arch, the anterior segment was distalized using two mini screws, and in the lower arch, the retraction was carried out using a sliding technique with reverse curve 17 × 25 SS wire and power chains so as to control the tipping of the upper and lower incisors (Figure 2). Class III elastics were utilized to improve the anchorage on the lower arch. Before the class III elastics were started, the patient was advised to undergo removal of all the third molars in the upper and lower arches. The goal of orthodontics treatment was to reduce the proclination of the upper and lower anterior teeth, close the diastema on the upper, make the space on the lower smaller for prosthodontic intervention, and achieve good occlusion. After treatment, restorations were placed on the three mandibular incisors.

### 2.4 Stage II: Gingival grafting

**FIGURE 3:**
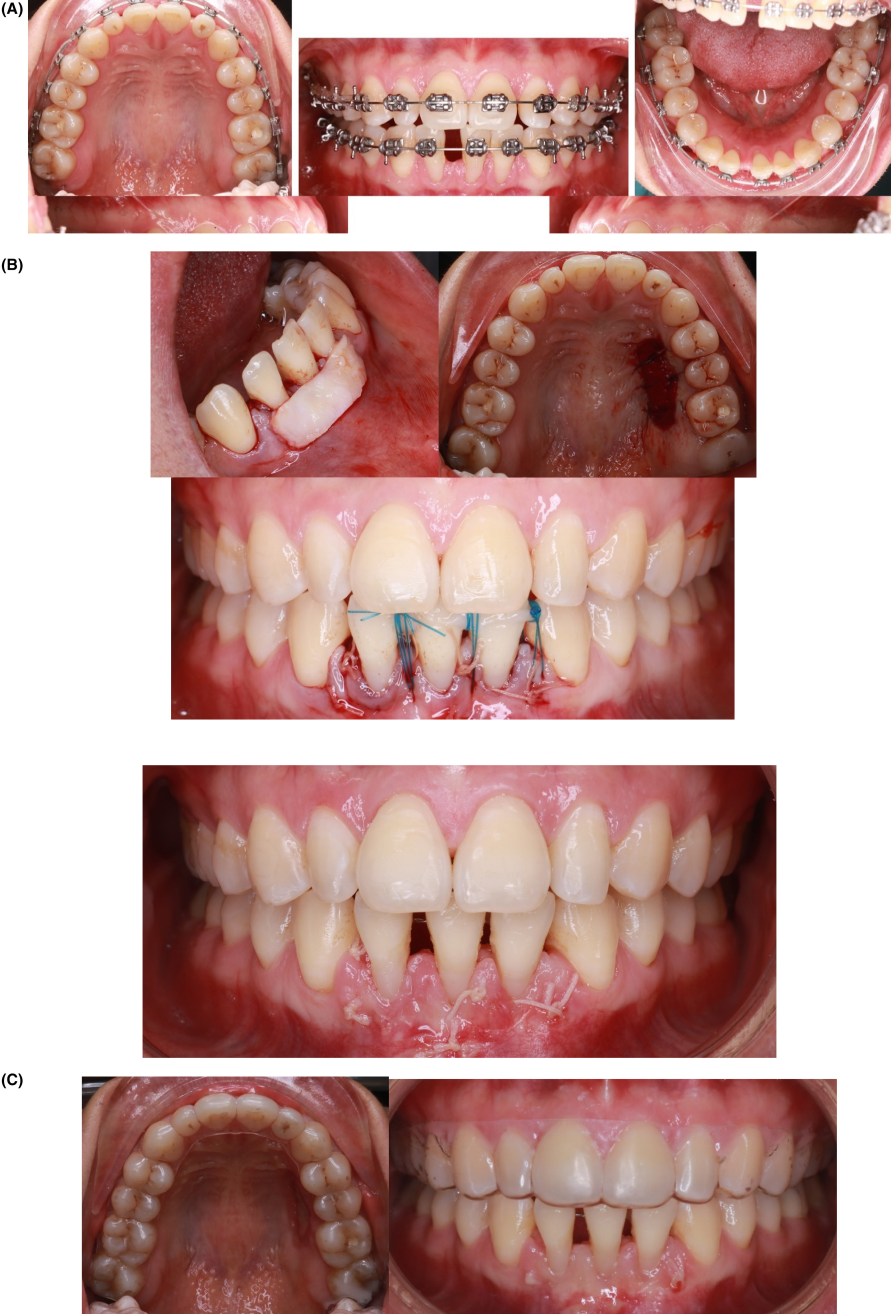
(A) Surgical grafting using subepithelial CTG. (B) Follow up after 2 weeks CTG. (C) Follow up after after 1 month.

After a 1‐month waiting period post debonding of the braces, gingival grafting was carried out using the subepithelial CTG technique (Connective tissue grafting tunnel technique) in the mandibular anterior region (Figure 3). The connective tissue graft was acquired from the right maxillary quadrant in the area mesial to the premolars and the first maxillary molar. 2% Lignocaine hydrochloride with adrenaline was used for local anesthesia, and then the first incision was given parallel to the long axis of the palate. The thickness of the flap was kept sufficient to ensure no tearing of the tissues. Root planning was done on the lower incisors before placement of the graft.

The harvested graft was sutured over the exposed root surfaces. Pressure was applied over the graft for a few minutes, and a periodontal pack was placed for healing. The patient was recalled after 2 weeks, the sutures were removed, and the healing appeared normal (Figure 3B,C).

### 2.5 Stage III: Restoration

**FIGURE 4:**
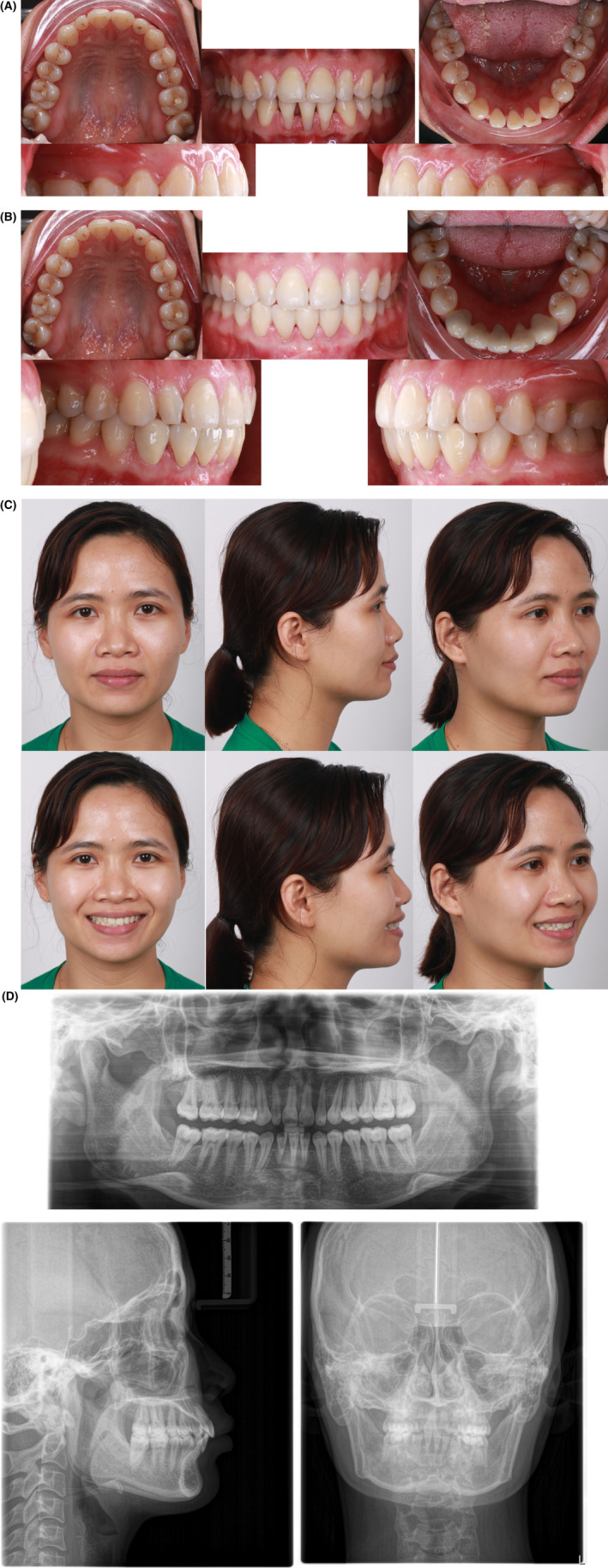
(A) Post‐treatment Intra oral Images of the patient. (B) After restorations. (C) Extra oral pictures post treatment. (D) Post treatment X rays.

After graft healing, restorations were placed in the mandibular arch from left to right lower canines.^3^, ^4^, ^5^ These were also intended to help close the diastema and provide good retention for the three lower incisors (Figure 4A,B,C).

## 3 TREATMENT RESULTS

All initial treatment objectives were achieved with a well‐aligned dentition, closure of all the diastemas, good occlusion, and improved facial esthetics.

**FIGURE 5:**
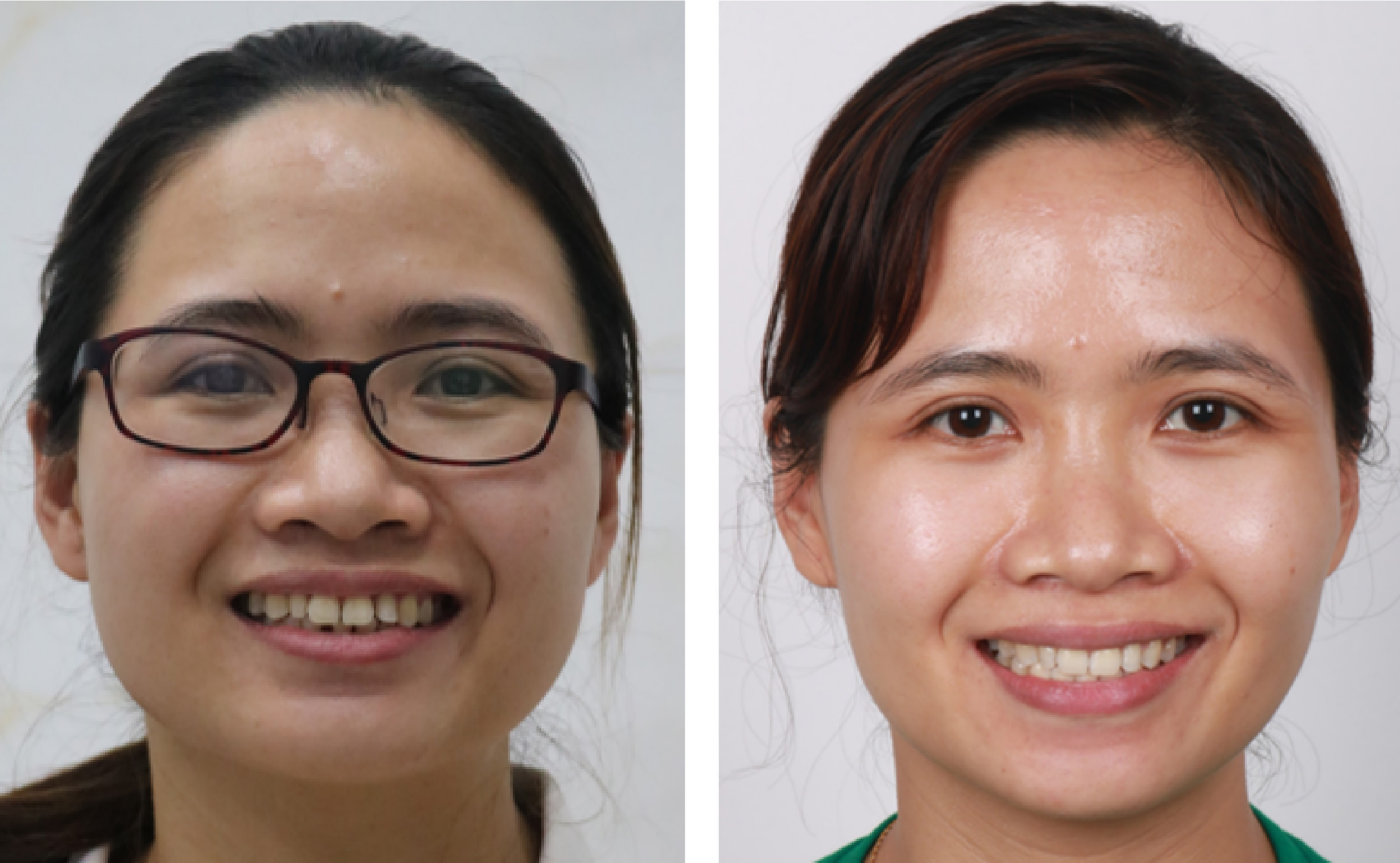
Improvement in the patient's smile.

The whole treatment was achieved by retraction of both upper and lower lips, resulting in a passive lip seal and relatively straight profile after treatment. Good root parallelism was achieved with minimal root resorption. There was an improvement in the incisor inclination and soft tissue profile, particularly across the E‐line and the nasolabial angle (Figure 4C,D and Figure 5).

## 4 DISCUSSION

In the current scenario, many treatment options are available to the patients for space closure and improving esthetics. Restorations and surgical grafts are some of the commonly available options that can drastically change the patient's smile.^6^, ^7^ These economical techniques can be carried out chairside, requiring much fewer appointments than other options. Dentists have utilized multidisciplinary approaches to ensure much better esthetic results than those obtained with singular approaches^8^, ^9^.

## 5 CONCLUSION

The present multidisciplinary approach combines fixed orthodontics, periodontal surgery, and prosthodontics to manage and improve a patient's smile and facial esthetics.

## AUTHOR CONTRIBUTIONS

**Hoang Viet:** Conceptualization; data curation; resources; software; writing – original draft; writing – review and editing. **Tran Hong Phuoc:** Conceptualization; resources; software; supervision; writing – original draft; writing – review and editing. **Hoang Ming Tuyen:** Conceptualization; resources; software; visualization; writing – original draft; writing – review and editing. **anand marya:** Conceptualization; formal analysis; supervision; writing – original draft; writing – review and editing.

## CONFLICT OF INTEREST STATEMENT

The authors report no conflict of interest.

## ETHICS STATEMENT

Because this report involves no experiment, ethics approval was not required.

## CONSENT

Written informed consent was obtained from the patient to publish this report in accordance with the journal's patient consent policy.

## Data Availability

Data related to the study can be provided on reasonable request.
